# Has Muslim Got Benefited from the National Health Mission? A Situational Analysis of Maternal Health Services in India

**DOI:** 10.4314/ejhs.v30i5.19

**Published:** 2020-09

**Authors:** Nasim Ahamed Mondal, Balhasan Ali, Md Illias Kanchan Sk

**Affiliations:** 1 Statistician, National Institute for Research in Reproductive Health, ICMR, Mumbai, India; 2 Doctoral Fellow, International Institute for Population Sciences, Mumbai, India; 3 International Institute for Population Sciences, Mumbai, India

**Keywords:** Maternal health, antenatal care, skilled birth attendants, postnatal care, Muslims, NFHS

## Abstract

**Background:**

It is a marked recognition that when the population is disaggregated by religion, wide disparities in the utilization of maternal health care services can be observed. The study was aimed to analyze the levels and trends of maternal health services among Muslims in India. The study also delineated the investigation of confounding factors attributed to maternal health services among the selected population.

**Methods:**

The study utilized the data from the third and fourth round of National Family Health Survey (NFHS), conducted in 2005–06 and 2015–16 respectively. The bivariate and multivariate logistic regression models were employed to accomplish the study objectives.

**Result:**

There is an increasing trend in the distributional patterns of all three indicators (full ANC, SBA and PNC) during the last two successive surveys. Muslim women belonging to Southern States were seen to be utilizing more maternal health care services as compared to Muslim women in the Northern States. Muslim populated States like Assam, Bihar, Jharkhand, Uttar Pradesh and West Bengal were far cry to achieve the MDG-15 target of utilization of 100 percent skilled birth attendants in 2015. Education, media exposure and wealth status appeared to be major confounding factors for determining the utilization of maternal health services.

**Conclusion:**

The study revealed that the utilizations of maternal health services among Muslims have progressed during the last decade. It can be concluded that the NHM policy has played an instrumental role in increasing the utilization of maternal health services among Muslims.

## Introduction

Every day, around 830 women die due to maternal or childbirth related complications around the world, and the resource limited countries account for almost 99 percent of all maternal deaths (1). The world witnessed more than 3,00,000 maternal deaths in 2015 (2). Almost 45,000 maternal deaths occurred in India, and the nation was recognized for the second home of maternal deaths after Nigeria (1). The level of maternal mortality ratio (MMR) in 2015 stood at 130 deaths per 100,000 live births which was far cry from 100 per 100,000 live births as per the Millennium Development Goal (MDG-5) for 2015 (3). After the failure to achieve maternal mortality ratio up to the expectations by three quarters between 1990 and 2015, the countries have now come under one roof towards a new goal. This goal is exhibited in Sustainable Development Goal 3 (SDG-3). The third sustainable development goal aimed to achieve less than 70 maternal deaths per 100,000 live births by 2030 (1). Approximately, 75 percent of maternal deaths in India occur due to direct obstetric causes such as haemorrhage, sepsis, hypertensive disorder, unsafe abortion and obstructed labor (4,5). The majority of these deaths can be prevented by proper and timely management of patients and providing extensive and effective maternal healthcare services (6). The utilization of maternal healthcare services varies widely among populations, and socioeconomically advanced population are availing relatively higher maternal healthcare facilities (7). It is a marked recognition that when the population is disaggregated by religion, wide disparities in the utilization of maternal healthcare services can be observed (8). The countries with the highest MMRs in the world are all parts of the Muslim world at large with the little exception (9).

However, social, economic and cultural differences are considered to be responsible for the low utilization of maternal healthcare services among Muslims (10). The researchers like Caldwell and Miller favoured the low education and low female autonomy to be the associated factors for substandard maternal care among Muslims (11) while some (12) have considered low standard of living and economic conditions responsible for the same.

Muslim population constituted 13.4 percent of the country's whole population (13). According to the NFHS-4 statistics, more than 13 percent of Indian women aged 15–49 years are Muslim (14). As per the Sachar Committee Report, the condition of Muslims in terms of economic, social and political status is on the margins and also lagging regarding numerous human development indicators in India (15). Whatever progress has been perceived in the light of utilization of maternal healthcare services is skewed in favour of few advanced socioeconomic groups in developing countries including India (16,17). Fertility level among Muslims is higher with relatively shorter birth interval (13). The short birth interval increases the risk on the health of mothers and the survival of their offspring. (18).

During the last few decades, India has witnessed several policies and programmes related to Maternal and Child Health (MCH) services. After the failure of optimum utilization of MCH services, the government of India launched National Health Mission (NHM) just before the NFHS-2 in the year of 2005 to improve the maternal and child healthcare services and to prevent the maternal mortality in the country. The NHM was also introduced in the country to bring down the variations in the maternal healthcare utilization among various groups. The effectiveness of the aforementioned programme has not been checked among the Muslim community in the previous research. The existing literature on Muslim women's healthcare reveals that Indian studies mainly focused on religious differences in fertility levels and contraceptive use while studies on socio-economic inequalities in maternal health services among Muslim women are rarely being observed (19). This research gap compelled us to engage in impromptu investigations of the factors responsible for maternal healthcare utilization among Muslims in India. This study was also designed to find out the status of maternal healthcare utilization in the preand post-NRHM periods.

## Materials and Methods

The study utilized the data from the third and fourth round of National Family Health Survey (NFHS), conducted in 2005–06 and 2015–16 respectively (14,20). The NFHS is nationally representative, an Indian version of Demographic Health Survey (DHS). The third and fourth round of NFHS covers more than 90,000 ever-married women aged 15–49 years from the entire nation. The NFHS is the prime source for the fertility, mortality, family planning, HIV-related knowledge, and important aspects of nutrition, health and healthcare services in the States as well as in the Nation. The survey also provides information relating to various socio-economic aspects of the respondents.

The NFHS-4 congregated information from 601,509 households. In the interviewed households, 723,875 eligible women aged 15–49 were identified for individual women's interviews. Interviews were completed with 699,686 women with a response rate of 97 percent. The NFHS-4 sample is a stratified two-stage sample. NFHS-3 covered 109,041 households. Interviews were conducted with 124,385 women aged 15–49. The urban and rural samples within each state were drawn separately. The sample within each state was allocated proportionally to the size of the state's urban and rural populations. A uniform sample design was adopted in all states.

The study considered place of residence (urban, rural), age group (15–24, 25–34, 35–49), birth order (1, 2, 3 and above), educational status (no education, primary education, secondary education, higher education), media exposure (no, yes), caste (scheduled caste, scheduled tribes, other backward castes (OBC), others, wealth index (poorest, poorer, middle, richer, richest) as the predictor variables.

This study used bivariate method to assess the distribution of full antenatal care, postnatal care, and skilled attendants at birth. Further, logistic regression analysis was employed to estimate the odds ratio with 95% CI for three critical maternal health indicators. For logistic regression analysis, we have taken the first category of predictor variables as a reference category.

The following operational definitions are used in this study

i. **Full antenatal care**: Full ANC was defined as a woman having four or more visits for ANC, having at least two tetanus injections and consuming 100 IFA (Iron and Folic Acid) tablets/syrup for their last birth.

ii. **Skilled attendants at birth**: The World Health Organization (WHO) defines a skilled birth attendant (SBA) as “an accredited health professional such as a midwife, doctor or nurse who has been educated and trained to proficiency in the skills needed to manage normal (uncomplicated) pregnancies, childbirth and the immediate postnatal period, and in the identification, management and referral of complications in women and newborns” (1).

iii. **Postnatal care**: PNC is the care given to the mother and her newborn baby immediately after the birth and for the first six weeks of life.

The study is based on secondary data analysis. No data was collected for this study. The data are available for free on the DHS website (https://dhsprogram.com/what-wedo/survey/survey-display-355.cfm). There is no need for ethical clearance.

## Results

Muslims belonging to the States like Kerala and Tamil Nadu had the distinction of achieving MDG-5 target of utilizing 100 percent skilled birth attendants. There were some major Muslim populated States like Assam, Bihar, Jharkhand Uttar Pradesh and West Bengal which were far cry to achieve the 100 percent Utilization of Skilled Birth Attendants (USBAs) in 2015.

Results show an increasing trend in the distributional patterns of all three indicators during the last decade (Table 1). The utilization of antenatal care did not show any significant increase during this period. Women belonging to Southern states (like Kerala, Tamil Nadu, Andhra Pradesh etc.) utilized more maternal healthcare services compared to Northern states (like Bihar, Madhya Pradesh and Chhattisgarh etc.). The result portrays that utilization of full antenatal care had been very low among all states except Kerala, Mizoram, and Tamil Nadu. Though utilization of full antenatal care had increased, the growth rate of increment in utilization of full antenatal care was significantly less during the decade. The results also show that Muslim women from Northeast States were using very low maternal health services. Utilization of postnatal care has also increased during the decade. However, the increment was less compared to skilled attendants at birth. Women belonging to Kerala were utilizing the highest percentage of full ANC (73.8 percent), SBA (100 percent) and PNC (91.9 percent) than any other states, whereas women belong to Nagaland (full ANC: 1.7 percent) and Meghalaya (SBA:25.6 percent; PNC:19.6 percent) were utilizing the lowest percentage of maternal health services in 2015–16.

**Table 1:** Percentage of Muslim women utilizing maternal health care services by State, India, 2005–06 and 2015–16

State	NFHS-3 (2005–06)			NFHS-4 (2015–16)		

	Full ANC	SBA	PNC	Full ANC	SBA	PNC
Andhra Pradesh	20.9	84.7	77.0	52.9	95.8	79.9
Arunachal Pradesh	5.6	15.4	9.4	2.4	68.4	48.4
Assam	1.2	13.9	6.4	9.8	59.2	36.8
Bihar	2.1	15.9	8.7	2.8	59.7	33.9
Chhattisgarh	2.2	72.9	58.5	22.9	95.0	81.5
Delhi	26.1	45.8	36.4	34.4	84.1	53.3
Goa	68.6	90.0	77.3	55.0	95.9	89.1
Gujarat	19.4	62.0	48.0	34.9	90.1	68.9
Haryana	1.2	16.5	10.7	5.0	52.3	35.0
Himachal Pradesh	20.2	71.7	26.3	18.4	82.0	61.9
Jammu and Kashmir	12.3	57.3	44.8	20.0	87.0	74.2
Jharkhand	3.9	30.8	15.1	6.4	68.4	42.1
Karnataka	26.8	76.2	60.6	27.8	91.5	64.7
Kerala	67.8	99.5	80.9	73.8	100.0	91.9
Madhya Pradesh	4.8	55.7	47.5	14.2	86.3	59.9
Maharashtra	11.1	78.2	66.5	25.9	91.3	75.2
Manipur	0.8	42.1	32.2	24.3	67.9	54.5
Meghalaya	0.0	20.7	19.6	7.4	25.6	19.6
Mizoram	33.3	100.0	100.0	40.7	89.3	83.9
Nagaland	0.0	20.7	11.3	1.7	35.1	23.9
Odisha	4.1	60.7	29.2	15.4	78.3	61.2
Punjab	8.1	50.7	27.1	23.0	81.0	77.1
Rajasthan	3.8	36.2	23.4	8.8	79.0	55.8
Sikkim	35.0	58.5	58.5	20.3	88.7	60.7
Tamil Nadu	41.8	100.0	99.1	44.2	99.9	68.5
Tripura	3.2	16.8	4.2	14.2	57.4	41.4
Uttar Pradesh	2.0	24.0	11.9	5.0	63.8	51.3
Uttarakhand	13.1	33.3	27.5	4.4	64.7	47.5
West Bengal	5.9	24.8	17.4	16.1	68.5	47.2
Total	9.3	38.7	29.1	17.1	73.6	54.8

Full ANC= Full ante-natal care; SBA= Skilled attendants at birth; PNC= Post-natal care

The results revealed that full antenatal care has increased by double in both rural and urban areas, reaching 25.3 percent in 20015–16, up from 14.2 percent in 2005–06 in the urban area; and in rural areas, it has reached 11.5 percent in the latest NFHS-4. Coverage of skilled attendants at birth among rural Muslim has increased very fast. The utilization of SBAs among rural Muslim women stood at 27 percent in 2005–06, and it increased 67 percent in 2015–16. Postnatal care utilization has also increased by about 47 percent in 2015–16 which was only 18 percent in 2005–06 among rural Muslim women. Although the result has shown that utilization of maternal healthcare services has been very low among 35–49 age group Muslim women, it has significantly increased among all age groups during this decade. Further, the results also portray that utilization of maternal healthcare has been decreasing with birth order but coverage has increased during the period. Education has a significant role in the utilization of maternal healthcare utilization. The results of the table show that only 3 percent in 2005–06 and 6 percent in 2015–16 Muslim women who had no education were utilizing maternal healthcare services in India. The results also revealed that with the increment of education, Muslim women were using more maternal health services, and utilization of maternal healthcare services has increased during the decade. The results also show that utilization of postnatal care decreased among Muslim women who had higher education during this decade. Media exposure also helped to increase the maternal health services among Muslim women. The results show that women who had media exposure were using more maternal health services than those women who had no media exposure. Further, higher caste women were utilizing more maternal health services in India. Although utilization of maternal healthcare services increased during the decade, the coverage of maternal healthcare services had been very less among scheduled caste women as compared to other social groups. The study also portrays that women belonging to the rich family were utilizing more maternal health services. Only 1.3 percent of poor women were seen to be utilizing full antenatal care compared to 29 percent of rich in 2005–06, and it increased only by 3 per cent during the decade. Further, the study also revealed that only 12 percent of poor women were using skilled attendant at birth compared to 83 percent of rich women in 2005–06 while only 5.5 percent poor were using postnatal care compared to 69 percent rich. The study shows that SBA and PNC increased among all strata of the society except full antenatal care during 2005–16.

The logistic regression model reveals that rural Muslim women were 0.89 (p<0.01) times, and 0.84 (p<0.001) times less likely to utilize full antenatal care and skilled attendants at birth respectively (Table 3). The results also show that with increasing age, Muslim women were more likely to utilize maternal healthcare services while with increasing birth order, women were less likely to utilize maternal healthcare services in India. Further, the results of the study revealed that education was also a significant determinant of maternal healthcare utilization in India among Muslim women. Compared to uneducated Muslim women, women who had higher education were 3.40 (p<0.001) times, 4.00 (p<0.001) times, and 1.79 (p<0.001) times more likely to utilize full antenatal care, skilled attendants at birth and postnatal care respectively. Media exposure also had a significant contribution to utilization of maternal health services and that women who had media exposure were 1.74 (p<0.001) times, 1.43 (p<0.001) times, and 1.37 (p<0.001) times more likely to utilize full antenatal care, skilled attendants at birth and postnatal care respectively. Further, the results indicate that rich women were utilizing more maternal healthcare services compared to the poor, and they were, 3.36 (p<0.001) times, and 3.26 (p<0.001) times more likely to utilize skilled attendants at birth, and postnatal care respectively in India.

**Table 3:** The logistic regression model showing the factors affecting the full antenatal care, skilled attendants at birth and postnatal care among Muslim women in India, 2015–16

Background Variables	Full ANC		SBA		PNC	

	O.R.	C.I. (95%)	O.R.	C.I. (95%)	O.R.	C.I. (95%)
**Place of Residence**						
Urban®						
Rural	0.89**	[0.81–0.97]	0.84***	[0.79–0.90]	0.96	[0.90–1.02]
**Age**						
15–24®						
25–34	1.24***	[1.12–1.36]	1.12**	[1.04–1.20]	1.26***	[1.17–1.35]
35–49	1.20*	[1.02–1.41]	1	[0.90–1.10]	1.16**	[1.05–1.29]
**Birth Order**						
1®						
2	0.86**	[0.78–0.95]	0.70***	[0.65–0.76]	0.78***	[0.72–0.84]
3 and above	0.64***	[0.57–0.72]	0.55***	[0.51–0.59]	0.59***	[0.55–0.65]
**Educational Status**						
No Education®						
Primary Education	1.34***	[1.15–1.55]	1.26***	[1.17–1.36]	1.08	[0.99–1.17]
Secondary Education	2.61***	[2.33–2.93]	2.08***	[1.94–2.23]	1.45***	[1.35–1.56]
Higher Education	3.40***	[2.88–4.01]	4.00***	[3.19–5.03]	1.79***	[1.54–2.09]
**Media Exposure**						
No®						
Yes	1.74***	[1.53–1.98]	1.43***	[1.35–1.52]	1.37***	[1.28–1.47]
**Caste**						
Scheduled Caste®						
Scheduled Tribe	4.11	[3.04–5.54]	1.45***	[1.23–1.71]	1.79***	[1.50–2.14]
OBC	1.62	[1.23–2.13]	1.06	[0.94–1.21]	1.29**	[1.11–1.48]
Others	1.74	[1.32–2.30]	0.97	[0.85–1.11]	1.23**	[1.06–1.42]
**Wealth Index**						
Poorest®						
Poorer	1.38	[1.15–1.66]	1.32***	[1.24–1.42]	1.48***	[1.36–1.61]
Middle	2.00	[1.66–2.40]	1.85***	[1.71–2.00]	2.07***	[1.88–2.27]
Richer	2.74	[2.28–3.29]	2.35***	[2.14–2.59]	2.66***	2.40–2.94]
Richest	3.47	[2.86–4.21]	3.36***	[2.96–3.81]	3.26***	[2.89–3.67]

Full ANC= Full ante-natal care; SBA= Skilled attendants at birth; PNC= Post-natal care

® Reference Category

*** p<0.001

** p<0.01

* p<0.05

## Discussion

Low utilization of maternal healthcare services among Muslim is not a new phenomenon. This issue has been positioned as a central debate for the public health scientist and demographers for several decades. The government of India has launched several policies and programs to address this issue. The National Health Mission (NHM) 2005 was a great endeavour of the Government of India to improve the maternal and child healthcare services and to reduce the gap among various religious groups in the country. Researches from national and global levels have tried to examine the low utilization of MCH services among Muslims compared to Hindus (12). The question has raised weather this NHM has played an instrumental role in increasing the maternal health services among Muslims. Therefore, this study is an attempt to picturize the role of NHM in maternal health services among Muslims.

This study is found that the Muslim women belonging to Southern States were utilizing more maternal healthcare services compared to Northern States. This finding also corroborated with previous studies (19). Another significant finding of this study is that the economically and educationally backward states like Bihar and Uttar Pradesh improved tremendously in the maternal health services which are not consistent the results of several existing studies (21,22). The Government's special attention may be the reason for this tremendous growth in the maternal health services in these States (23). The results also highlighted that the Muslim populated states have manifested enormous enhancement in the maternal health services during the last decade. This result is the manifestation of the acceptance of NHM policies among Muslims.

The study also investigated the various confounding factors associated with a high level of maternal health services among the Muslim population. The factors like education, media exposure and wealth were positively associated with maternal health services. Educated Muslim women were more likely to utilize all three maternal health indicators compared to uneducated women (12,24,25). Women who had media exposure were 1.74 times, 1.43 times and 1.37 times more likely to utilize full antenatal care, skilled attendants at birth and postnatal care respectively (12,25,26). Rich women were utilizing more maternal healthcare services compared to the poor; this finding is also supported by many researchers (24,27). Birth order was negatively associated with the maternal health services in the study (28). This negative association can be the outcome of safe delivery experiences (29).

The study shows significant growth in the utilisation of maternal health services during the last two successive demographic health surveys. The educational attainment, media exposure, place of residence, wealth status and birth order appeared as confounding factors in the study. Finally, it can be concluded that the NHM policy played an instrumental role in increasing the utilization of maternal health services among Muslims. This study has provided a new dimension in the field of public health research that proper implementation of policies and plan of action may be proven as betterment for the backward group of society.

## Figures and Tables

**Figure 1:**
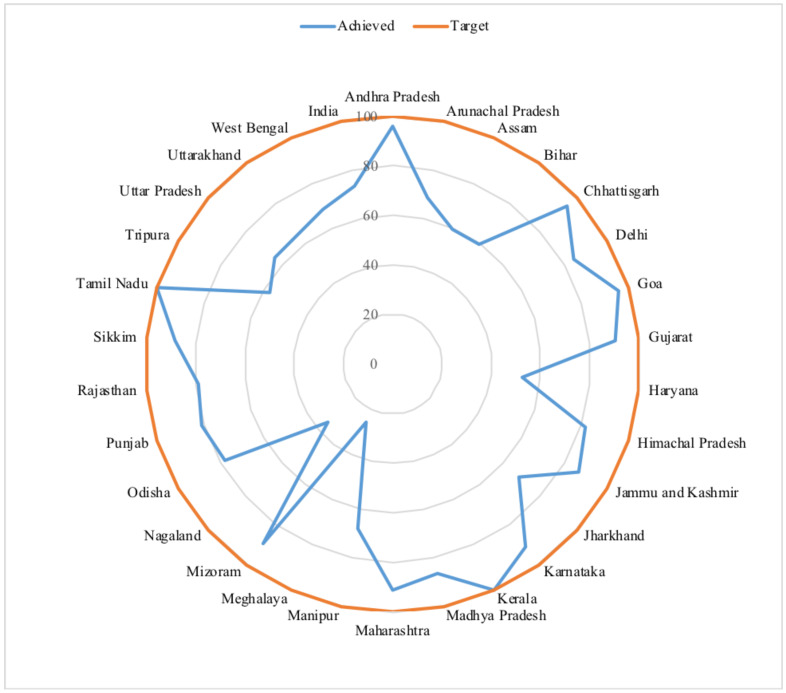
Utilization of skilled birth attendants in Indian states by MDG, 2015 (Source: National Family Health Survey round 4, 2015–16 and United Nations, The Millennium Development Goals Report 2015)

**Table 2:** Percentage of Muslim women utilizing maternal health care services with selected background characteristics, India, 2005–06 and 2015–16

Background Variables	NFHS-3 (2005–06)			NFHS-4 (2015–16)		

	Full ANC	SBA	PNC	Full ANC	SBA	PNC
**Place of Residence**						
Urban	14.2	63.1	50.6	25.3	84.2	65.7
Rural	6.9	27.0	18.4	11.5	66.6	47.3
**Age group**						
15–24	10.1	42.8	32.1	18.2	77.5	56.4
25–34	10.0	38.4	29.3	17.8	73.9	56.2
35–49	3.7	26.0	19.0	10.9	60.7	44.6
**Birth Order**						
1	16.5	58.8	58.8	22.0	83.5	64.7
2	13.0	45.6	45.6	20.9	76.7	58.8
3 and above	5.1	26.7	26.7	11.2	63.1	45.5
**Educational Status**						
No Education	2.6	21.9	12.9	6.1	58.6	40.1
Primary Education	8.2	40.5	26.3	11.9	69.6	48.0
Secondary Education	21.6	71.6	58.2	25.1	86.3	66.1
Higher Education	39.3	94.9	88.4	39.1	95.5	78.7
**Media Exposure**						
No	1.9	19.7	11.9	6.3	56.6	36.5
Yes	13.7	51.0	39.3	22.2	82.4	63.4
**Caste**						
Scheduled Caste	0.8	22.8	12.9	8.8	67.5	42.0
Scheduled Tribe	7.1	27.1	20.7	12.5	70.2	48.4
OBC	9.8	39.6	29.2	17.9	73.1	55.9
Others	9.4	40.1	31.8	16.9	73.5	53.5
**Wealth Index**						
Poorest	1.3	12.3	5.5	4.8	52.2	30.9
Poorer	3.4	21.5	13.0	9.1	66.5	44.6
Middle	6.7	35.4	24.0	16.6	78.9	59.2
Richer	15.1	66.0	50.1	25.9	87.1	69.2
Richest	28.5	83.3	69.2	34.2	92.6	76.5

Full ANC= Full ante-natal care; SBA= Skilled attendants at birth; PNC= Post-natal care

## References

[1] World Health Organization (2015). Trends in maternal mortality 1990–2015 estimates from WHO, UNICEF, UNFPA, World Bank Group and the United Nations Population Division executive summary.

[2] Alkema L, Chou D, Hogan D, Zhang S, Moller AB, Gemmill A, Fat DM, Boerma T, Temmerman M, Mathers C, Say L (2016). Global, regional, and national levels and trends in maternal mortality between 1990 and 2015, with scenario-based projections to 2030: a systematic analysis by the UN Maternal Mortality Estimation Inter-Agency Group. The Lancet.

[3] Sample Registration System (SRS) (2018). Special bulletin on maternal mortality in India (2011–13).

[4] Zaman S, Begum A (2014). Maternal mortality at a rural medical college of Assam: a retrospective study. J Obstet Gynaecol Barpeta.

[5] Sample Registration System (SRS) (2015). Special bulletin on maternal mortality in India (2011–13).

[6] Martin N, Montagne R (2017). The last person you'd expect to die in childbirth.

[7] Yaya S, Bishwajit G, Shah V (2016). Wealth, education and urban-rural inequality and maternal healthcare service usage in Malawi. BMJ global health.

[8] Sanneving L, Trygg N, Saxena D, Mavalankar D, Thomsen S (2013). Inequity in India: the case of maternal and reproductive health. Global health action.

[9] Liese KL, Maeder AB (2018). Safer Muslim motherhood: Social conditions and maternal mortality in the Muslim world. Global public health.

[10] Rutaremwa G, Wandera SO, Jhamba T, Akiror E, Kiconco A (2015). Determinants of maternal health services utilization in Uganda. BMC health services research.

[11] Caldwell J, McDonald P (1982). Influence of maternal education on infant and child mortality: levels and causes. Health policy and education.

[12] Das A, Mohanty PC, Haque MM (2016). Case on Indian Muslim Mother's Healthcare Utilisation: Its Patterns, Trends and Comparison. Asia-Pacific Journal of Management Research and Innovation.

[13] Census of India 2011 (2011). Office of the Registrar General & Census Commissioner, India.

[14] International Institute for Population Sciences (2007). National Family Health Survey (NFHS-3), 2005–06 India.

[15] Sachar R, Hamid S, Oommen TK, Basith MA, Basant R, Majeed A, Shariff A (2006). Social, economic and educational status of the Muslim community of India. East Asian Bureau of Economic Research.

[16] Haque M (2009). Individual's characteristics affecting maternal health services utilization: married adolescents and their use of maternal health services in Bangladesh. The Internet Journal of Health.

[17] Dalal K, Shabnam J, Andrews-Chavez J, Mårtensson LB, Timpka T (2012). Economic empowerment of women and utilization of maternal delivery care in Bangladesh. International Journal of Preventive Medicine.

[18] Raj P (2005). Pregnancy complications and health-seeking behavior among Married women in Uttar Pradesh, India. Res Practice Social Sci.

[19] Ali B, Dhillon P, Mohanty SK (2019). Inequalities in the utilization of maternal health care in the pre-and post-National Health Mission periods in India. Journal of Biosocial Science.

[20] International Institute for Population Sciences (IIPS), ICF (2017). National Family Health Survey (NFHS-4) 2015–16.

[21] Paul VK, Sachdev HS, Mavalankar D, Ramachandran P, Sankar MJ, Bhandari N, Sreenivas V, Sundararaman T, Govil D, Osrin D, Kirkwood B (2011). Reproductive health, and child health and nutrition in India: meeting the challenge. The Lancet.

[22] Ram F, Ram U, Singh A (2010). Future demand for maternal and child health services from public health facilities in Uttar Pradesh. Journal of Family Welfare.

[23] O'Neil S, Naeve K, Ved R (2017). An examination of the maternal health quality of care landscape in India. Mathematica Policy Research.

[24] Kumar A, Singh A (2015). Explaining the gap in the use of maternal healthcare services between social groups in India. Journal of Public Health.

[25] Mukherjee S (2014). Determinants of maternal care utilization among young Muslim women in India. International Journal of Public Health Research.

[26] Rabbi AF (2012). Mass media exposure and its impact on fertility: Current scenario of Bangladesh. Journal of Scientific Research.

[27] Pathak PK, Singh A, Subramanian SV (2010). Economic inequalities in maternal health care: prenatal care and skilled birth attendance in India, 1992–2006. PloS one.

[28] Kifle D, Azale T, Gelaw YA, Melsew YA (2017). Maternal health care service seeking behaviors and associated factors among women in rural Haramaya District, Eastern Ethiopia: a triangulated community-based cross-sectional study. Reproductive Health.

[29] Ononokpono DN, Odimegwu CO (2014). Determinants of maternal health care utilization in Nigeria: a multilevel approach. The Pan African Medical Journal.

